# Disorder of Sexual Development Males With XYY in Blood Have Exactly X/XY/XYY Mosaicism in Gonad Tissues

**DOI:** 10.3389/fgene.2021.616693

**Published:** 2021-04-12

**Authors:** Yongjia Yang, Fang Chen, Zhenqing Luo, Yu Zheng, Jiayong Zheng, Yuyan Fu, Weijian Chen, Haiyan Luo

**Affiliations:** ^1^The Laboratory of Genetics and Metabolism, Hunan Children’s Research Institute, Hunan Children’s Hospital, University of South China, Changsha, China; ^2^Key Laboratory of Obstetrics and Gynecology, Wenzhou People’s Hospital, Wenzhou, China; ^3^Department of Pathology, Hunan Children’s Hospital, University of South China, Changsha, China; ^4^Center for Diagnosis and Treatment of Rare Diseases, Hunan Children’s Hospital, University of South China, Changsha, China

**Keywords:** XYY syndrome, DSD, gonad biopsy, FISH, exome sequencing, mosaicism

## Abstract

Y chromosome represents masculinization. The extra Y chromosome of XYY patients usually leads to over-masculinization phenotypes. The occurrence of several DSD cases with XYY in blood is controversial. Is XYY associated with disorder of sex development (DSD)? What is the mechanism behind DSD in males with XYY in blood? To this end, this study retrospectively analyzed blood-karyotype data of 4,437 DSD male children and karyotypes data of 6,259 newborn males as the control. Exome sequencing (ES) was performed to test whether the patients with DSD and with XYY in blood had other variants on known DSD-genes. Testicular biopsy was performed. Fluorescence *in situ* hybridization (FISH) was used to test whether a sex chromosome mosaicism was present in the oral epithelial cells or gonad tissue of patients with DSD and with XYY in blood. Among 4,437 DSD males who received cytogenetic evaluation, 14 patients with 47,XYY were identified. By contrast, five individuals among the 6,259 controls had 47,XYY. XYY in blood is more frequent among males with DSD than in other males (*p* = 0.004). The XYY karyotypes were confirmed again by GTG-banding in blood samples and by FISH performed on oral epithelial cells. ES on seven XYY DSD patients was successfully performed, but results did not identify any pathogenic variant on 55 known DSD genes. Gonad biopsy (*n* = 3) revealed testicular dysplasia and true hermaphroditism. FISH of gonad tissues (*n* = 3) showed that all of the samples had mosaic for X/XY/XYY. This study is the first to investigate the relationship between XYY in blood and DSD. The knowledge that XYY is in the blood and in oral cells have X/XY/XYY mosaicism in gonadal tissue is new for both researchers and clinicians who seek to understand the genetic basis of DSD males.

## Introduction

The diagnosis of chromosome diseases is highly dependent on GTG-binding of blood samples. The 47,XYY syndrome is a common sex chromosome aneuploidy that occurs in 1 out of 1,000 male births (Bardsley et al., 2013; Gao et al., 2014; Jo et al., 2016; Yao et al., 2019). Although XYY is diagnosed routinely through GTG-banding of peripheral blood, the phenotypes of XYY

patients may vary greatly, ranging from no phenotype and relatively few abnormalities to multi-systemic symptoms; for a specific symptom, the severity can vary among individuals (Kim et al., 2013; Bardsley et al., 2013). Some scholars believe that XYY is associated with a status of over-masculinization, because the existence of an extra Y chromosome and XYY individuals usually results in tall stature, impulsivity, and/or sex organ overdevelopment (macroorchidism and macropenis) (Bardsley et al., 2013; Jo et al., 2015). Several reports stated that some men institutionalized for antisocial behavior were found to have an increased frequency of the XYY karyotype, and males in prison with XYY had higher testosterone than healthy age-matched controls (Hook, 1973; Schiavi et al., 1984). For decades, the claim that XYY males tend to exhibit more aggressive, anti-social, and criminal behavior than XY males is controversial, but this hypothesis has never been substantiated (Lenroot et al., 2009).

Another more pointed argument was the opposite of over-masculinization; XYY has also been sporadically reported in connection with several cases of disorder of sex development (DSD) (Boczkowski, 1970; Grace and Campbell, 1978; Rivera et al., 1979; Terada et al., 1984; Okamoto et al., 1988; Diego Nuñez et al., 1992; Suzuki et al., 1999; Benasayag et al., 2001; Monastirli et al., 2005; Bardsley et al., 2013; Latrech et al., 2015). Such DSD phenotypes of XYY patients include micropenis, testicular dysplasia, true-hermaphrodite, and complete sex reversal (Supplementary Table 1) (Boczkowski, 1970; Grace and Campbell, 1978; Rivera et al., 1979; Terada et al., 1984; Okamoto et al., 1988; Diego Nuñez et al., 1992; Suzuki et al., 1999; Benasayag et al., 2001; Monastirli et al., 2005; Bardsley et al., 2013; Latrech et al., 2015). However, due to the fact that all previously reported DSD XYY patients are sporadic cases, and the DSD frequency is as high as 1/200 in a general male population, it is still unknown whether DSD is associated with XYY or just the coincidence of XYY and DSD. Given that more than half of patients with DSD can be traced to a pathogenic variant on one of 55 known genes related to DSD (Eggers et al., 2016; Wang et al., 2017), it is also possible that the DSD phenotypes are the consequence of an extra Y chromosome and a pathogenic variant on one of the known DSD genes.

## Materials and Methods

### Ethics Statement

The study protocol was approved by the Academic Committee of Hunan Children’s Hospital (Approval number: HCHLL58, Changsha City, Hunan Province, China). All participants or their parents provided written informed consent to partake in this study.

### Patient Clinical and Karyotype Data

Identifying and diagnostic information and karyotype data were obtained from the records of all males diagnosed with DSD from Hunan Children’s Hospital (Changsha City, China) from July 2010 to June 2018. DSD was evaluated and diagnosed by pediatric urologists. Karyotype data of neonatal umbilical cord blood from Wenzhou People’s Hospital (Wenzhou City, China) were used as the control karyotype data. These data included a total of 10,086 karyotype data of the general population, of which 6,259 are males, obtained from May 2012 to April 2018.

### Cytogenetic Analysis

Peripheral venous blood was collected in a vacutainer sodium heparin vial. Slides were prepared from phytohemagglutinin-stimulated peripheral lymphocyte cultures by using standard cytogenetic methods. Giemsa (GTG) banding at a 400-band level to a 550-band level was performed in accordance with the standard laboratory protocol. Two different cultures, corresponding to two different series of slides from each sample, were separately prepared and analyzed. At least 40 metaphases were analyzed for each individual. For the second round of GTG-banding evaluation, 100 metaphases were analyzed per patient.

### Exome Sequencing

Genomic DNA (200 ng) of each individual was sheared by Biorupter (Diagenode, Belgium) to acquire 150–200 bp fragments. The ends of DNA fragment were repaired and Illumina Adaptor was added (Fast Library Prep Kit, iGeneTech, Beijing, China). After constructing a sequencing library, the whole exons were captured with AIExome Enrichment Kit V1 (iGeneTech, Beijing, China) and sequenced on Illumina platform (Illumina, San Diego, CA, United States) with 150 base-paired end reads. Raw reads were filtered to remove low quality reads by using FastQC. Then, clean reads were mapped to the reference genome GRCh37 by using Bwa. After removing duplications, SNV and InDel were called and annotated by using GATK. 11.9 G bases were obtained for each sample. The average yield was ∼16.6 Gb with an error rate of <0.1%. Furthermore, >80% bases had a Phred quality score of ≥30 (Q30).

### Gonad Biopsy, Hematoxylin and Eosin Stain (H&E), and Fluorescence *in situ* Hybridization

Gonadal biopsy was performed for three DSD children with XYY. H&E was performed routinely for all available tissue specimens according to standard procedures. Fluorescence *in situ* hybridization (FISH) analyses were performed on oral epithelial cells and on gonad tissues. The centromere satellite probes Xp11.1-q11.1 Alpha Satellite DNA Vysis CEP X (DXZ1) Spectrum Green Probe and Yp11.1-q11.1 Alpha Satellite DNA Vysis CEP Y (DYZ3) Spectrum Orange Probe were purchased from Abbott-Vysis company (Abbott Park, IL, United States).

## Results

### Disorder of Sex Development Males Have an High Frequency of XYY

From July 2010 to June 2018, 4,437 DSD males received genetic and clinical evaluation in Hunan Children’s Hospital. Of these 4,437 DSD males, 3,885 were simplex with one phenotype or deformity and 552 were complex phenotypes with more than one phenotype. Among the 3,885 simplex DSD male children, 2,717 had hypospadias (accounting for 61.24% of overall DSD), 897 had penis dysplasia (20.22%), 107 had cryptorchidism (2.41%), and 93 had hypoplastic testis (2.10%), whereas 71 had other conditions (1.60%). The 552 complex DSD children presented the following: (i) two or more DSD phenotypes (*n* = 459) (10.34%); and (ii) DSD phenotype and other malformations, such as developmental delay, skeletal deformities, and others (*n* = 93) (2.10%).

Among 4,437 males with DSD, 136 (3.07%) were affected by cytogenetic abnormalities (Supplementary Table 2), including 47,XXY (*n* = 28), 45,X/46,XY (*n* = 21), 47,XYY (*n* = 14), 46,XX (*n* = 12), 46,XY/46,XX (*n* = 8), 46,XY,del(9p22) (*n* = 6), different chromosome translocation (*n* = 17), and others (*n* = 30). Therefore, XYY was one of major chromosome abnormalities in DSD males.

The clinical data for 14 male children with DSD and XYY are provided in Table 1. Blood endocrine test was performed for eight of the 14 patients with DSD and XYY (Supplementary Table 3), among which two patients exhibited pituitary prolactin above the normal (27.47 and 19.86; the normal range for children was 1–19 pg/ml).

**TABLE 1:** Clinic information for 14 DSD males with XYY in blood, which observed from 4,437 DSD children.

**ID**	**Age^a^**	**Karyotype in blood**	**Genital abnormalities**	**Other phenotypes**
A56	0–5	47,XYY	Penile dysplasia and absent right testis	Intellectual disability
A57	0–5	47,XYY	Penile and testicle dysplasia	Intellectual disability and bilateral strabismus
A58	0–5	47,XYY	Penile dysplasia; penis adduction; and supra urethral dehiscence	Intellectual disability and short stature
A59	5–10	47,XYY	Penile dysplasia and penis adduction	–
A60	0–5	47,XYY	Penile dysplasia; penile flexion deformity; hypospadias; and testis dysplasia	–
A61	0–5	47,XYY	Hypospadias and scrotum division	Epilepsy
A62	5–10	47,XYY	Penile dysplasia and testis dysplasia	–
A63	5–10	47,XYY	Hypospadias and penile scrotal translocation deformity	–
A64	0–5	47,XYY	Hypospadias; penile flexion deformity; and testis dysplasia	–
A65	0–5	47,XYY	Penile dysplasia and hypospadias	Right ectopic kidney
A66	0–5	47,XYY	Hypospadias, testis dysplasia; penile dysplasia; and absent right testis	Female reproductive tissues observed
A67	0–5	47,XYY	Hypospadias; penile dysplasia; and penis adduction	–
A68	5–10	47,XYY	Penile dysplasia; penis adduction; and testis dysplasia	Intellectual Disability
A69	5–10	47,XYY	Hypospadias and penile dysplasia	–

*^a^Year range.*

The karyotype data of neonatal umbilical cord blood were used as the control for this study. In 6,259 male controls, a total of 51 individuals had abnormal karyotype (Supplementary Table 4), and among them, five individuals had XYY (phenotypes were unavailable). This result was consistent with the reported finding that the XYY frequency in the general male population was 1/1,000 (Bardsley et al., 2013; Jo et al., 2016).

Between-group comparison showed significant difference in the frequency of XYY in controls (5/6,259, 0.08%) and in the DSD group (14/4437, 0.32%) (*p* = 0.004). If only complex DSD was considered, then the frequency of XYY in complex DSD (14/552, 2.54%) was even higher than that in the control group (5/6259, 0.08%) (*p* < 0.001).

### Repeating GTG Banding Assay on Blood and FISH on Oral Cells Did Not Support Sex Chromosome Aneuploid Mosaicism for Patients With DSD

To test if sex chromosome aneuploid mosaicism was present in the 14 patients with DSD (the first round of GTG banding reported as 47,XYY; 40 metaphase cells were analyzed per patient), we intended to re-visit all of them to replicate the GTG banding. However, only 10 patients were followed up, and peripheral venous blood samples were obtained from seven individuals (listed in Supplementary Table 5). The GTG banding of blood was performed again for these seven patients. For each one, 100 metaphase cells were analyzed. None of these seven patients with DSD presented with sex chromosome aneuploid mosaicism. All have true 47,XYY in blood.

We further collected oral epithelial cells from these seven patients with XYY and DSD and carried out FISH tests on them by using the centromere satellite probes of the Y and X chromosomes. The oral cells from all seven patients (oral epithelial cells) have two signals of the Y chromosome and one signal of the X chromosome (Supplementary Figure 1).

### None of Seven DSD-XYY Patients Harbor Pathogenic Variant on 55 Known DSD Genes

DSD has 1/200 chance of occurrence among general males, and more than half of DSD males have pathogenic variants on known DSD genes (Eggers et al., 2016). The XYY frequency in a male population is about 1/1,000 (Supplementary Table 4) (Bardsley et al., 2013; Jo et al., 2016). It is possible for a patient with DSD to have XYY aneuploidy and a DSD-gene mutation. To test this hypothesis, exome sequencing (ES) was performed for seven patients with DSD and with XYY in blood (for those whose genomic DNA data were available). ES was performed, and an average of 10.51 Gb data were obtained within the target region of each sample. The quality statistics for ES are shown in Supplementary Table 5.

We focused on 55 known causative genes for male DSD (Supplementary Table 6) (Eggers et al., 2016). We used the following filtering steps: (i) rare variants (MAF < 0.005, gnomAD_Eas); (ii) variants absent in in-house controls (201 ES data of males without DSD; parts are published before (Yang et al., 2019; Zhu et al., 2019); and (iii) considering damaging variants (loss-of-function and damaging missense variants) (Yang et al., 2019). Only one variant remained, namely, chr17:77753158, C to T (CBX2,NM_032647:exon3:c.117-3C > T) on A61 (Supplementary Figure 2A). Results of literature search showed the existence of a girl with 46,XY who harbored compound heterozygous variants on CBX2 (Biason-Lauber et al., 2009).

Read map of ES data for CBX2 was re-checked, and the whole CBX2 coding regions were covered (>30×) by ES for A61, but no other variant was identifiable (data not shown). We intended to test the pathogenicity for the CBX2 variant through segregation analysis of the family. Results showed that the CBX2-c.117-3C > T variant was shared by his unaffected grandfather (Supplementary Figure 2). Therefore, the significance of the variant CBX2-c.117-3C > T is unknown. Altogether, no direct evidence supported the assumption that XYY patients harbored a DSD gene pathogenic variant, which co-contributed to DSD.

### Gonad Biopsy and H&E Staining

**FIGURE 1:**
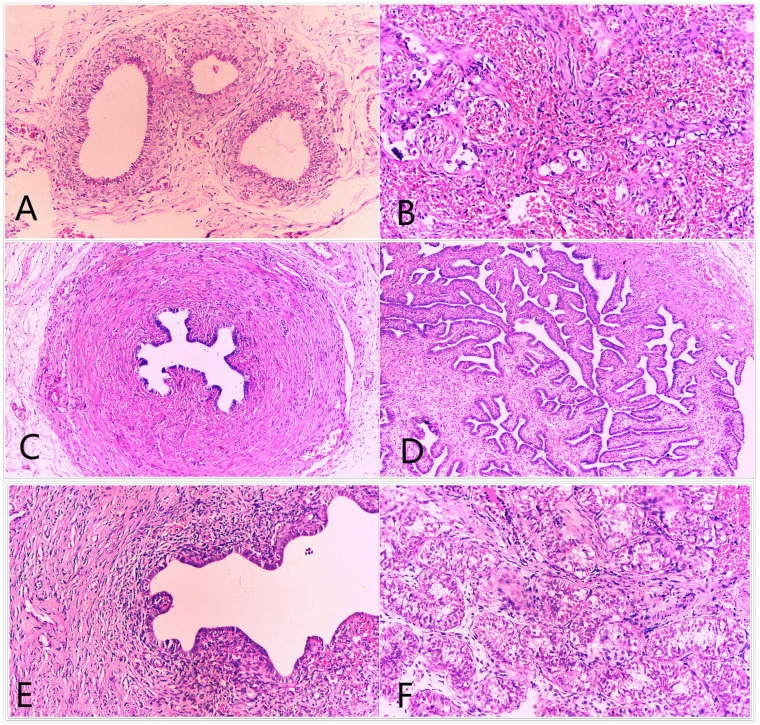
H&E staining of the biopsy gonad tissues from a severest DSD patient with XYY in blood (A66). For A66, surgical exploration of scrotum and groin revealed **(A)** the structure of epididymis, **(B)** the structure of ovary (primodial follicles are visible), **(C)** the structure of vas deferens, **(D)** the structure of fallopian tubes, and **(E)** structure of uterus. For A66, right testis biopsy revealed the dysplasia of spermatogenic tubules **(F)**.

The pathogenic analysis of gonad tissue may give clues to elucidate the mechanism of the development of DSD in XYY males. Most patients with DSD and with XYY in blood were subjected to urological surgery for deformity correction. Three of them (or their guardians) agreed to undergo gonad biopsy. H&E staining of the biopsy tissue showed that 3/3 DSD-XYY patients exhibited dysplasia of spermatogenic tubules of testicles (two bilateral; one unilateral) (Figure 1F and Supplementary Figure 3). In one of the three (1/3) DSD-XYY patients (A66), only the right testicle was observed in the process of operation (data not shown).

Through laparoscopic, ureteroscopic, and cystoscopic examinations, fragmentary gonad tissues were found and excised. H&E staining of the biopsied tissues showed the structures of bisexual reproductive organ-tissues, including epididymis, ovary, vas deferens, fallopian tubes, and uterus (Figures 1A–E).

### Fluorescence *in situ* Hybridization on Gonad Tissues Revealed X/XY/XYY Mosaicism

**FIGURE 2:**
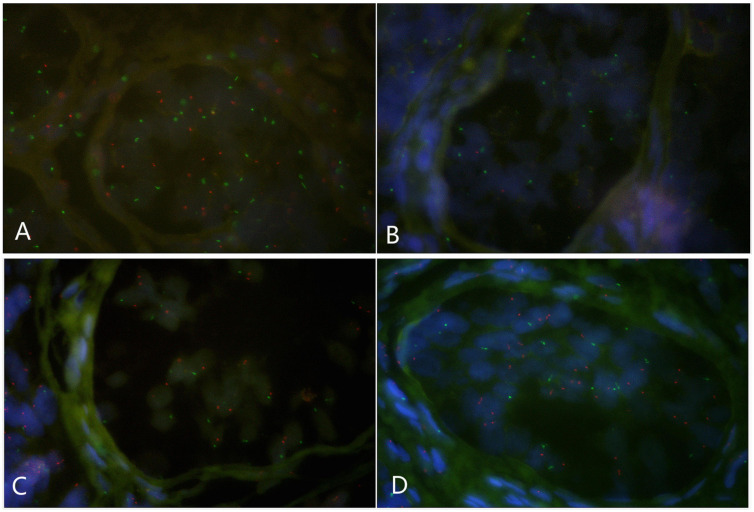
Fluorescence *in situ* hybridization (FISH) on spermatogenic tubules (green spot: FISH centromeric signals of Chromosome X; red spot: signals of Chromosome Y). **(A)** A spermatogenic tubule from a normal testicle (NC1553), 23 green and 22 red spots in the tubule implicates XY; BCD: Spermatogenic tubules from A58 testes. In panel **(B)**, 17 green and 2 red signals detected implicates X/XY mosaicism. In panel **(C)**, 17 green and 19 red signals detected implicates XY. In panel **(D)**, 17 green and 34 red signals detected implicates XYY.

**FIGURE 3:**
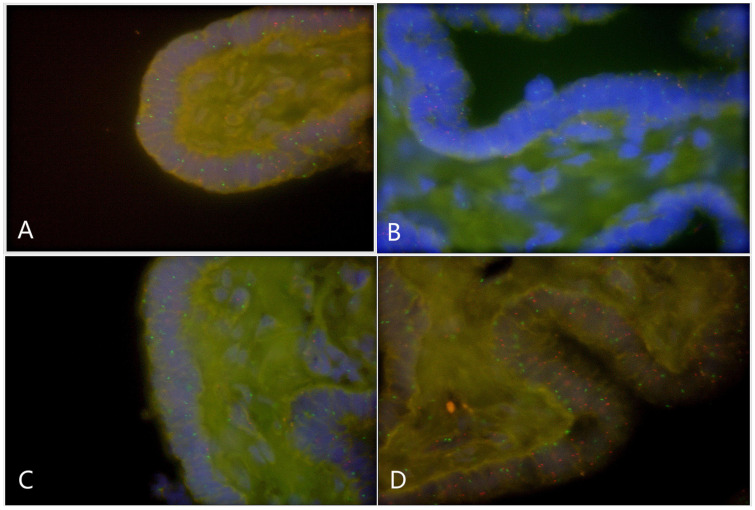
Fluorescence *in situ* hybridization (FISH) on fallopian tubes tissue that originated from a DSD boy with XYY in blood (A66) (green spot: FISH centromeric signals of Chromosome X; red spot: signals of Chromosome Y). **(A)** X/XY signals; **(B)** X/XY signals; **(C)** X/XY signals; **(D)** XYY signals.

Paraffin embedded sections showed incomplete nucleus of the tissue cells. For the evaluation of chromosome aneuploidy, analyzing centromeric signal of FISH by using single cells as a unit on slides would be unreasonable. We then intended to evaluate the centromeric signal by using spermatogenic tubules as a unit. Indeed, in a normal testicle donated by a boy that died due to a traffic accident (NC1553), we found that the X or Y signal per tubule are basically equal (Figure 2A, Table 2, and Supplementary Figure 4). FISH results on spermatogenic tubules of three patients with DSD (A58,A66 and A60; their karyotypes are 47,XYY in blood) indicated that all of their testicular tissues have X/XY/XYY mosaicism, although the mosaic percentage varied (Figures 2A–D, Table 2, and Supplementary Figure 4). For patient A66, FISH was performed for the fallopian tube tissue, and the results also indicated X/XY/XYY mosaicism (Figure 3).

**TABLE 2:** Fluorescence *in situ* hybridization (FISH) signal counts per spermatogenic tubules for one normal control (NC1553) and three DSD children with XYY in blood (A58, A66, and A60).

**NC 1553**	**Green**	**Red**	**Green/red**	**Predicted karyotype**	**A58**	**Green**	**Red**	**Green/red**	**Predicted karyotype**	**A66**	**Green**	**Red**	**Green/red**	**Predicted karyotype**	**A60**	**Green**	**Red**	**Green/red**	**Predicted karyotype**
Tub1	16	15	1.07	XY	Tub1	22	44	0.50	XYY	Tub1	22	4	5.5	X/XY	Tub1	13	0	–	X
Tub2	31	35	0.89	XY	Tub2	10	21	0.48	XYY	Tub2	27	10	2.7	X/XY	Tub2	25	4	6.25	X/XY
Tub3	29	28	1.04	XY	Tub3	14	28	0.50	XYY	Tub3	33	11	3	X/XY	Tub3	27	8	3.38	X/XY
Tub4	14	17	0.82	XY	Tub4	18	6	3	X/XY	Tub4	15	1	15	X/XY	Tub4	31	7	4.43	X/XY
Tub5	18	19	0.95	XY	Tub5	10	11	0.91	XY	Tub5	19	2	9.5	X/XY	Tub5	27	5	5.40	X/XY
Tub6	16	14	1.14	XY	Tub6	41	92	0.45	XYY	Tub6	37	19	1.95	X/XY	Tub6	17	3	5.67	X/XY
Tub7	25	25	1.00	XY	Tub7	27	50	0.54	XYY	Tub7	40	18	2.22	X/XY	Tub7	22	0	–	X
Tub8	14	14	1.00	XY	Tub8	26	48	0.54	XYY	Tub8	9	9	1.00	XY	Tub8	22	2	11.00	X/XY
Tub9	11	11	1.00	XY	Tub9	43	44	0.98	XY	Tub9	39	11	3.55	X/XY	Tub9	19	7	2.71	X/XY
Tub10	12	13	0.92	XY	Tub10	39	74	0.53	XYY	Tub10	19	2	9.50	X/XY	Tub10	33	2	16.50	X/XY
Tub11	12	11	1.09	XY	Tub11	20	20	1.00	XY	Tub11	16	3	5.33	X/XY	Tub11	27	9	3.00	X/XY
Tub12	17	16	1.06	XY	Tub12	15	16	0.94	XY	Tub12	29	0	–	X	Tub12	35	6	5.83	X/XY
Tub13	13	13	1.00	XY	Tub13	17	2	8.5	X/XY	Tub13	17	0	–	X	Tub13	16	4	4.00	X/XY
Tub14	19	22	0.86	XY	Tub14	28	51	0.55	XYY	Tub14	29	4	7.25	X/XY	Tub14	15	3	5.00	X/XY
Tub15	28	31	0.90	XY	Tub15	22	4	5.5	X/XY	Tub15	22	3	7.33	X/XY	Tub15	24	4	6.00	X/XY

*Tub = spermatogenic tubule, Green = green spot signal of X centromere; Red = red spot signal of Y centromere.*

## Discussion

Since 1963, at least 23 Patients with DSD with the karyotype of XYY in blood have been reported (Supplementary Table 1) (Boczkowski, 1970; Grace and Campbell, 1978; Rivera et al., 1979; Terada et al., 1984; Okamoto et al., 1988; Diego Nuñez et al., 1992; Suzuki et al., 1999; Benasayag et al., 2001; Monastirli et al., 2005; Bardsley et al., 2013; Latrech et al., 2015). Phenotypes for these patients with DSD are variable, e.g., female-external-genitalia, bisexual appearances, or hypospadias (Supplementary Table 1) (Boczkowski, 1970; Grace and Campbell, 1978; Rivera et al., 1979; Terada et al., 1984; Okamoto et al., 1988; Diego Nuñez et al., 1992; Suzuki et al., 1999; Benasayag et al., 2001; Monastirli et al., 2005; Bardsley et al., 2013; Latrech et al., 2015). Combining the well-characterized knowledge of XYY leading to over-masculinization (macroorchidism, macropenis, and tall stature) and information on DSD in XYY patients, scientists and clinical practitioners tend to assume that XYY leads to bipolarized sex determination.

However, because all DSD XYY patients in the literature are sporadically reported cases, and DSD frequency in general males is as high as 1/200 (the coincidence of XYY and a DSD? Latrech et al. (2015) 14), a clear evidence of XYY’s association with DSD is lacking. To address this issue, we compared the XYY frequencies of 4,437 patients with DSD and 6,259 newborn-general males. Statistical results indicated a solid association between XYY in blood and male DSD (*p* = 0.004).

Given that the condition of more than 50% of patients with DSD can be explained by pathogenic variant on one of the 55 known DSD genes (Eggers et al., 2016), another possibility exists, i.e., the DSD phenotypes in XYY patients are due to the coincidence of XYY and a DSD gene mutation. To test this coincidence, we analyzed the coding regions of 55 known male DSD genes in seven patients with DSD and with XYY in blood by next generation sequencing. However, we did not identify any definite pathogenic variants on these 55 genes in patients with DSD with XYY in blood.

To further explore the mechanism underlying DSD in males with XYY in blood, gonad biopsy was performed on three patients. H&E staining of gonad tissues revealed the presence of dysplasia in spermatogenic tubules in all three patients and bisexual reproductive organ in one of the three patients. The FISH test results revealed the occurrence of X/XY/XYY mosaic in gonad tissues for all three patients with DSD and with XYY in blood.

One limitation of this study is that the genetic analysis (including but not limited to the gonad FISH test) did not involve adult infertile men with XYY in blood. Previously studies have shown that the majority of individuals with XYY in blood are fertile because of the loss of the extra Y before meiosis (Rives et al., 2005), and the remaining patients with XYY in blood are infertile (Suzuki et al., 1999; Borjian Boroujeni et al., 2017). However, the mechanism that explains why a number of XYY men are infertile is not fully understood. This limitation can be compensated by a recent study (Sciurano et al., 2019) carried out by Sciurano et al., who described a 35-year-old man with primary infertility. The man had hypotrophic testes (10 and 12 ml testicular volume; the normal volume is more than 15 ml) and inguinal hernia during childhood; Yq12 FISH detected X/XY/XYY mosaicism (50% XYY; 44%XY; 6%X, respectively) in Sertoli cells (Sciurano et al., 2019).

In conclusion, we identified X/XY/XYY mosaicism in gonad tissues and detected the lack of sex chromosome mosaicism on oral epithelial cells on patients with DSD and with XYY in blood. These findings explain the DSD phenotypes in patients with XYY in blood and also indicate the uncharacterized proliferation-mode of gonad cells. Further study is needed to explore such novel cell proliferative mode.

## Data Availability Statement

The datasets presented in this study can be found in online repositories. The name of the repository and accession number can be found below: National Center for Biotechnology Information (NCBI) ClinVar, https://www.ncbi.nlm.nih.gov/clinvar/, VCV000996035.1

## Author Contributions

HL, YY, and WC designed the research, analyzed the experimental data, and wrote the manuscript. YY, FC, ZL, YZ, JZ, YF, WC, and HL performed the sample collection and the research. All authors contributed to the article and approved the submitted version.

## Conflict of Interest

The authors declare that the research was conducted in the absence of any commercial or financial relationships that could be construed as a potential conflict of interest.
