# Supplementary material for: Disorder of Sexual Development Males With XYY in Blood Have Exactly X/XY/XYY Mosaicism in Gonad Tissues
Source: Front Genet. 2021 Apr 12;12:616693. doi: 10.3389/fgene.2021.616693 (PMC8072476; doi:10.3389/fgene.2021.616693)

Supplementary Table 1: Representative DSD individuals with XYY in blood reported in literatures. a: M= male; b: Y=year, M=month,D=day.

Publications	n	Sex	Age	DSD related phenotypes	Other phenotypes
Rivera et al., 1979	1	M	5Y10M	Micropenis, hypospadias<br>Right testis regression	-
Terada et al., 1984	1	M	29Y	Right undescended testis	-
E. Okamoto et al., 1988	2	M	-	Hypospadias, cryptorchism	-
Diego et al., 1992	2	M	-	Cryptorchism, puberty delay	-
Suzuki et al., 1999	1	M	11M	Bilateral cryptorchism	-
Monastirli et al., 2005	1	M	6Y	Cryptorchism, gynecomastia	-
Bardsley et al., 2013	1/90<br>2/90<br>5/90	M	-	Hypospadias<br>Cryptorchism<br>Inguinal hernia	-
Latrech et al, 2015	2	M	3M	Hypospadias, ectopic testis	-
		M	1M	Micropenis, ectopic testis	Congenital heart defect
Boczkowski et al., 1970	2	F	-	Eunuchoidal body proportions,Female external genitalia,<br>Lack of breast development, Primary amenorrhea	Overheight
		F	-	Eunuchoidal body shape, Short vagina, No uterus, Inguinal hernias,<br>May be testis on the right, No gonads on the left and Scoliosis	Overheight, Scoliosis
Grace et al., 1978	1	F	1Y2M	Hypoplastic external genitalia, No uterus and Short stature	Short stature
Suzuki et al., 1999	1	M	11M	Bilateral cryptorchism	-
Benasayag et al., 2001	1	F	16Y	Female external genitalia, Lack of breast development, Primary amenorrhea, Short vagina, No ovaries or uterus, Bilateral immature testes, Right-sided inguinal hernia, SRY gene positive	Overheight

Supplementary Table2: Cytogenetic abnormalities detected by GTG-banding in 4437 DSD male children in present study.

Karyotype	Total
46,XY,del(9)(p23)	
46,XY,del(9)(p23)	
46,XY,del(9)(p24)	
46,XY,del(9)(p22)	
46,XY,del(9)(p23)	
46,XY,del(6)(p23)	6
45,X[10]/46,XY[90]	
45,X[18]/46,XY[32]	
45,X[7]/46,XY[25]	
45,X[11]/46,XY[19]	
45,X[7]/46,XY[48]	
45,X[12]/46,XY[21]	
45,X[39]/46,XY[27]	
45,X[22]/46,XY[11]	
45,X[11]/46,XY[22]	
45,X[9]/46,XY[13]	
45,X[7]/46,XY[82]	
45,X[14]/46,XY[7]	
45,X[9]/46,XY[41]	
45,X[8]/46,XY[16]	
45,X[16]/46,XY[44]	
45,X[10]/46,XY[50]	
45,X[8]/46,XY[72]	
45,X[18]/46,XY[41]	
45,X[23]/46,XY[36]	
45,X[12]/46,XY[42]	
45,X[20]/46,XY[15]	21
47,XXY	
47,XXY	
47,XXY	
47,XXY	
47,XXY	
47,XXY	

47,XXY	
47,XXY	
47,XXY	
47,XXY	
47,XXY	
47,XXY	
47,XXY	
47,XXY	
47,XXY	
47,XXY	
47,XXY	
47,XXY	
47,XXY	
47,XXY	
47,XXY	
47,XXY	
47,XXY	
47,XXY	
47,XXY	
47,XXY	
47,XXY	
47,XXY	28
47,XYY	
47,XYY	
47,XYY	
47,XYY	
47,XYY	
47,XYY	
47,XYY	
47,XYY	
47,XYY	
47,XYY	
47,XYY	
47,XYY	
47,XYY	
47,XYY	14
46,XX[53]/46,XY[17]	

46,XY[19]/46,XX[21]	
46,XX[37]/46,XY[9]	
46,XX[13]/46,XY[17]	
46,XX[95]/46,XY[5]	
46,XX[12]/46,XY[19]	
46,XX[40]/46,XY[20]	
46,XY[12]/46,XX[11]	8
46,XX	
46,XX	
46,XX	
46,XX	
46,XX	
46,XX	
46,XX	
46,XX	
46,XX	
46,XX	
46,XX	
46,XX	12
46,XY,t(18;19)(q23;p12)	
46,XY,t(4;18)(p14;p11)	
46,XY,t(5;7)(q22;q32)	
46,XY,t(5;13)(q34;q12)	
46,XY,t(1;2)(p34;q37)	
46,XY,t(5;6)(q14;q22)	
46,XY,t(3;9)(p21q23)	
46,XY,t(1;9)(q42;p22)	
46,XY,t(8;10)(q24.1;q26)	
46,XY,t(1;10)(p10;q10)	
46,XY,t(11;22)(p11;q11)	
46,XY,t(4;3)(q12;q34)	
46,XY,t(5;9)(q13;q22)	
46,XY,t(3;4)(p14;q22)	
46,XY,t(3;10)(p21;q26)	
46,XY,t(4;11)(q31;p12)	
46,XY,t(1;8)(p32;q24)	17

45,X[21]/46,X,+mar[11]/47,X,+2mar[6]	
45,X[18]/46,X,+mar[18]/47,X,+2mar[1]	
45,X[6]/46,XY[76]/46,X,+mar,-Y[16]	
45,Xp+,-Y[24]/46,Xp+,-Y,+mar[9]	
45,X[11]/46,X,-Y,+mar[14]	
46,X,+mar[50]/45,X[10]	
45,X[12]/46,X,+mar[10]	
46,X,+mar[20]/45,X[10]	
46,XY[11]/47,XYY[23]	
45,X[10]/46,X,+mar[11]	
45,X[8]/46,X,+mar[14]	
45,X[17]/46,X,+mar[22]	
46,XY,+mar,inv(Y)(P11q11),22ps+	
47,XY,+mar[12]/46,XY[48]	
47,Xp+?,Yqh-*2	
46,X,YP+,15p+	
46,XY,-5,+der(5)t(5;7)(p15;q21)mat	
46,X,dic(Y)(p11)[56]/45,X[4]	
46,X,dic(Y)[25]/45,X[7]	
47,XY,+i(Xq10)	
46,XY,9p+,6q-(?)	
45,X[24]/46,X,psu dic(Y)(p11)[26]	
46,XY[15]/47,XYY[11]	
46,XY,del(16)(q21)[10]/46,XY[49]	
46,XY,del(16)(q21)[9]/46,XY[36]	
45,X[27]/46,X,Yq-[9]	
46,XY,del(13)(q33)	
46,XY,del int(3)(q26.3)	
48,XXXY	
48,XXYY	30
	136

Supplementary Table 3: Available results of blood endocrine test for 8/14 DSD XYY patients. FSH: Follicle Stimulating Hormone, LH: Luteinizing Hormone; E2: Estradiol II; TTT: Testosterone; PRL: Prolactin

Note: the red scores represent out of the normal range. Normal range for children: FSH(0.4-5.8 mIU/ml); LH(0-2.3 mIU/ml); E2(0-39 pg/ml); TTT(0-43 pg/ml); PRL(1.0-19 pg/ml).

ID	Age	FSH	LH	E2	TTT	PRL
A58	0-5 years	3.37	0.38	15.2	4.31	27.47
A67	0-5 years	NA	NA	12.6	NA	18.29
A60	0-5 years	1.19	1.01	12	2.06	11.01
A62	5-10 years	3.09	0.11	21	2.03	6.23
A63	0-5 years	3.18	0.31	14	2.48	16.08
A64	5-10 years	2.43	0.9	16	1.02	15.79
A65	0-5 years	2.56	0.14	14	0.25	7.75
A68	5-10 years	1.19	0.02	17	2.15	19.86

Supplementary Table 4: Chromosome abnormalities in 6259 general male controls. Note: GTG-banding performed using samples of neonatal umbilical cord blood.

Karyotypes	Case count
46, XY, inv(1)(q22q32)	1
46, XY, inv(7)(q22q32)	1
46, XY, inv(6)(p111q13)	1
46, XY, inv(7)(q22q36)	1
46, XY, inv(10)(p112q22)	1
46, XY, inv(10)(p12q23)	1
46, XY, inv(11)(p15q23)	1
46, XY, inv(19)(p13q13)	1
46, XY, t(1;3)(p36;p12)	1
46, XY, t(1;9)(p36;q33)	1
46, XY, t(1;18)(q32;q21)	1
46, XY, t(1;22)(p22;q12)	1
46, XY, t(2;12)(q21;q22)	1
46, XY, t(3;22)(p22;q13)	1
46, XY, t(4;8)(q21;p12)	1
46, XY, t(4;14)(q33;q13)	1
46, XY, t(5;10)(q13;q11.2)	1
46, XY, t(6;13)(q11.2;q22)	1
46, XY, t(6;17)(q23;q21)	1
46, XY, t(10;15)(p13;q11)	1

46, XY, t(12;16)(q24;p13)	1
46, XY, t(15;17)(q14;q12)	1
46, XY, t(17;22)(q21;q11)	1
45, XY, der(13;14)(q10;q10)	4
46, XY, der(13;15)(q10;q10)	1
46, XY, del(5)(p14)	1
47, XY, +mar/46, XY	1
47, XXY/46, XY	2
47, XY, +21/46, XY	1
47, XY, +21	6
47, XY, +mar	1
47, XXY	6
47, XYY	5
Total:	51

Supplementary Fig 1: Fluorescence in situ hybridization (FISH) on oral epithelial cells for 7 DSD patients with XYY in blood.

A: A representative FISH signal picture of A oral epithelial cells. Green spots: centromere of chromosome X; Red spots: centromere of chromosome Y; Yellow spot: centromere of chromosome 18

B: Summery data of green and red signals for each patient. G=Green signal; R=Red signal.

A:

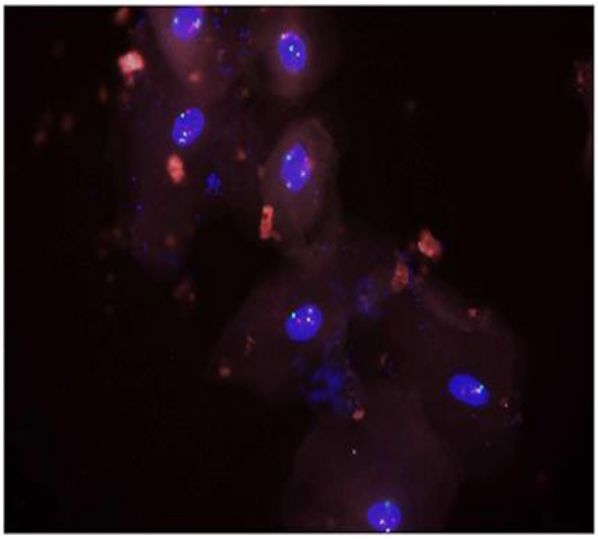

B:

ID	1G2R	1G1R	2G1R	2G2R	Cells analyzed
A1962	99	1	0	0	100
A5511	100	0	0	0	100
A7676	100	0	0	0	100
B732	99	0	1	0	100
B1138	100	0	0	0	100
B2756	100	0	0	0	100
B7015	100	0	0	0	100

S Table 5: Exome sequencing data quality for 7 DSD patients with XYY in blood.

Sample	Clean bases(Mb)	Total reads mapping rate(%)	Target mean depth	Coverage rate(%)	T 20X coverage rate(%)
A65	10865.93	97.54	128.5	99.9	99.05
A57	8804.62	99.73	113.73	99.88	98.84
A58	9868.93	99.71	124.94	99.89	98.86
A60	9083.48	99.7	117.01	99.89	98.86
A61	10689.09	99.66	111.1	99.88	98.74
A64	12676.75	99.67	132.57	99.89	99.09
A66	11569.23	99.68	129.59	99.88	99.03

S Table 6: List of 55 known causative genes for male DSD. Note: this table according to reference 14. AR=autosomal recessive; AD=autosomal dominant; XL=X linked; dup=duplication.

	Gene	Locus	OMIM	Inheritance
1	CBX2	17q25.3	602770	AR
2	DHH	12q13.12	605423	AR,AD
3	DMRT1	9p24.3	602424	AD:deletion
4	DMRT2	9p24.3	604935	AD:deletion
5	GATA4	8p23.1	600576	AD
6	NR0B1	Xp21.2	300473	XL-dup
7	NR5A1	9q33.3	184757	AD
8	MAP3K1	5q11.2	600982	AD
9	SOX9	17q24.3	608106	AD
10	SRY	Yp11.2	480000	AD
11	TSPYL1	6q22.1	604714	AR
12	WNT4	1p36.12	603490	AD:dup
13	WT1	11p13	607102	AD
14	ZFPM2	8q23.1	603693	AD
15	AKR1C2	10p15.1	600450	AR

16	AKR1C4	10p15.1	600451	AR
17	AMH	19p13.3	600957	AR
18	AMHR2	12q13.13	600956	AR
19	AR	Xq12	313700	XL
20	ARX	Xp21.3	300215	XL
21	ATRX	Xq21.1	300032	XL
22	CDKN1C	11p15.4	600856	AD
23	CYB5A	18q22.3	613218	AR
24	CYP11A1	15q24.1	118485	AR
25	CYP17A1	10q24.32	609300	AR
26	CYP19A1	15q21.2	107910	AR
27	FGFR2	10q26.13	176943	AD
28	HSD17B3	9q22.32	605573	AR
29	HSD3B2	1p12	613890	AR
30	LHCGR	2p16.3	152790	AR
31	POR	7q11.23	124015	AR
32	SRD5A2	2p23.1	607306	AR
33	STAR	8p11.23	600617	AR
34	BBS9	7p14.3	615986	AR
35	CHD7	8q12.2	608892	AD
36	FGF8	10q24.32	612702	AD
37	FGFR1	8p11.23	147950	AD
38	FSHB	11p14.1	136530	AD
39	GNRH1	8p21.2	152760	AR
40	GNRHR	4q13.2	138850	AR
41	HESX1	3p14.3	601802	AD
42	KAL1	Xp22.31	300836	XL
43	KISS1R	19p13.3	604161	AD
44	LEP	7q32.1	164160	AR
45	LHX3	9q34.3	600577	AR

46	PROK2	3p13	607002	AD
47	PROKR2	20p12.3	607123	AD
48	PROP1	5q35.3	601538	AR
49	TAC3	12q13.3	162330	AR
50	WDR11	10q26.12	606417	AD
51	ATF3	1q32.3	603148	AD
52	HOXA13	7p15.2	142959	AD
53	INSL3	19p13.11	146738	AD
54	MAMLD1	Xq28	300120	XL
55	RXFP2	13q13.1	606655	AD

Supplementary Fig 2:

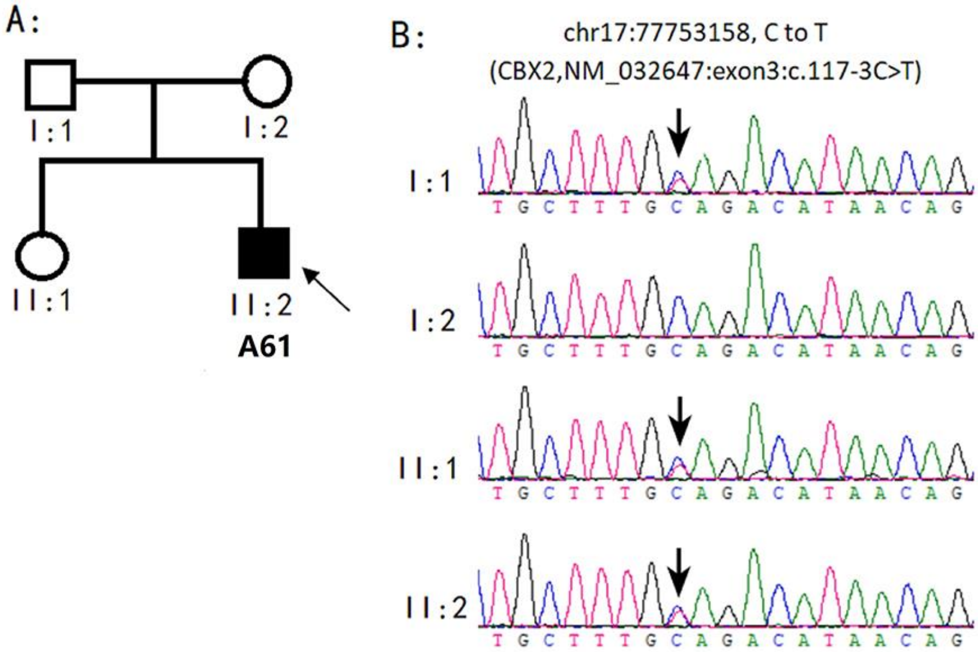

C:

Chromosome	Start	End	Exon	Reads number	Depth
chr17	77751976	77752106	CBX2:exon1	8024	61.2519
chr17	77752182	77752226	CBX2:exon2	5333	118.511
chr17	77753160	77753226	CBX2:exon3	4768	71.1642
chr17	77755494	77755600	CBX2:exon4	12369	115.598
chr17	77755494	77756331	CBX2:exon4	67963	81.1014
chr17	77757530	77761449	CBX2:exon5	135068	34.4561

Supplementary Fig 3: Testicular biopsy and H&E staining (200×). AB: left and right testes for A60; CD: left and right testes for A58.

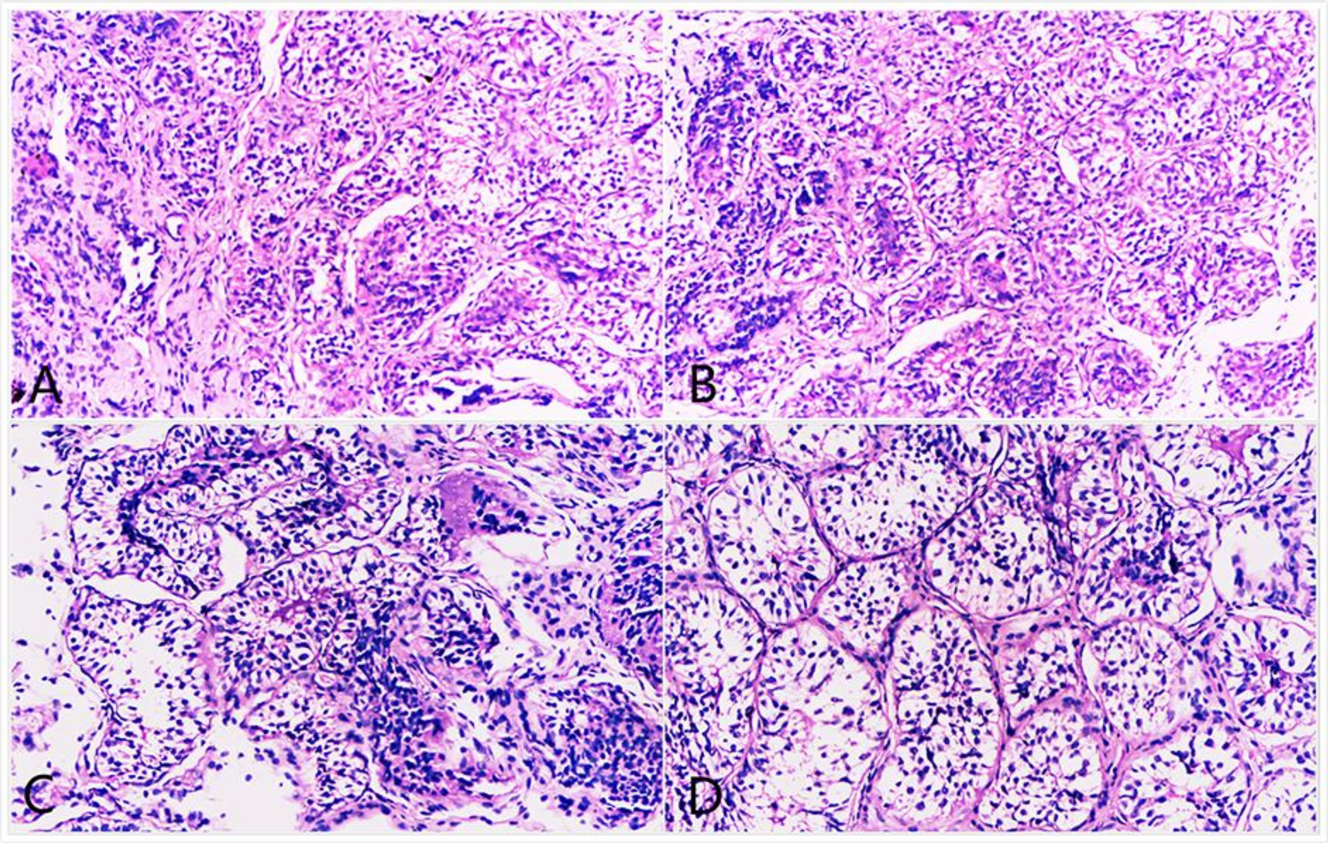

Supplementary Fig 4: FISH on spermatogenic tubules (Green spot: centromeric signal of Chromosome X; Red spot: centromeric signal of Chromosome Y). ABC: Tubules from a normal testicle of individual 1533. DEF: Tubules from testicle of patient A7676, represent X, X/XY, X/XYY respectively. GHI: Tubules from testicle of patient A60, represent three X, X/XY/XYY respectively.

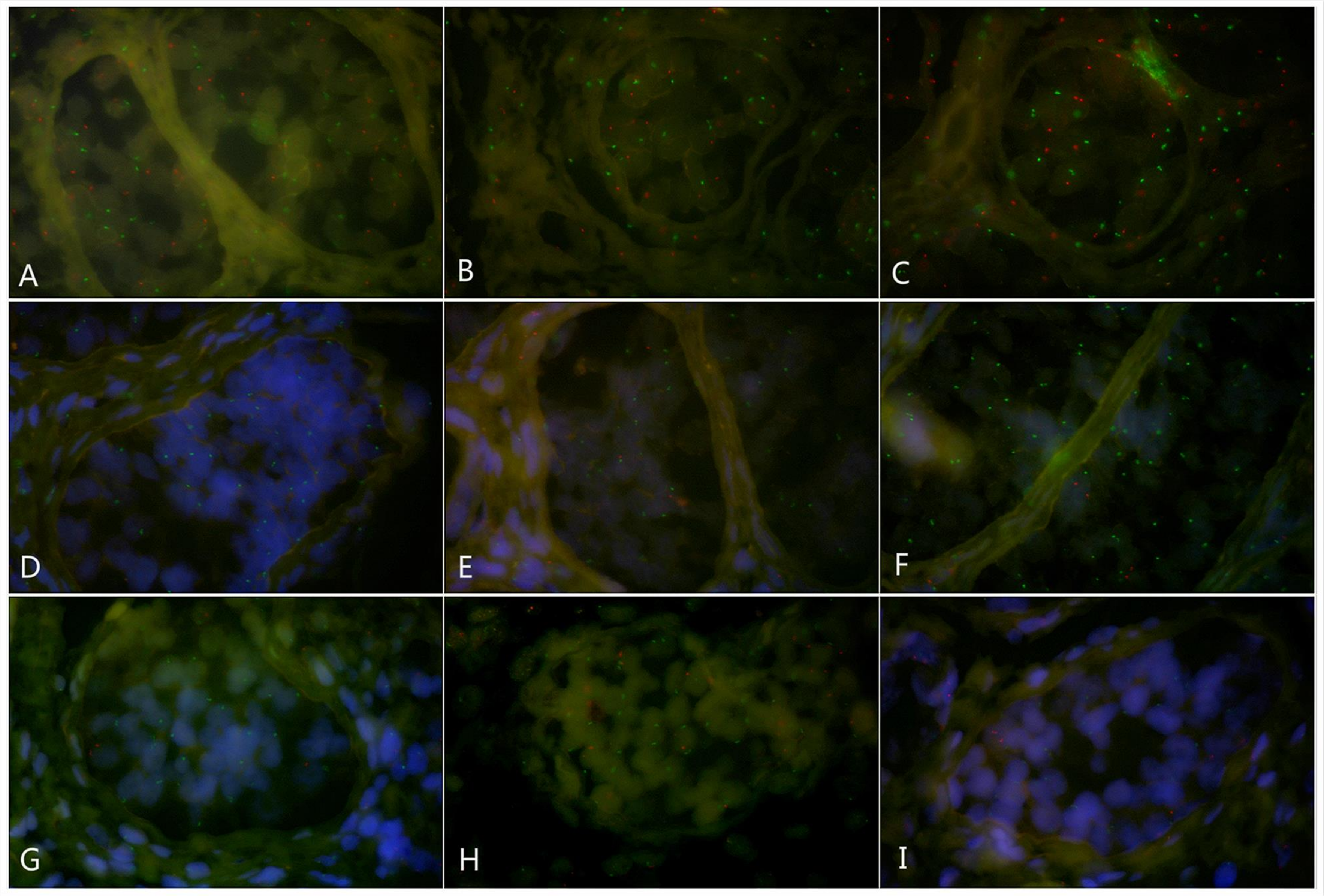

Supplement: Supplementary file 1 [file Data_Sheet_1.PDF]
